# Acceptability of a Personally Controlled Health Record in a Community-Based Setting: Implications for Policy and Design

**DOI:** 10.2196/jmir.1187

**Published:** 2009-04-29

**Authors:** Elissa R Weitzman, Liljana Kaci, Kenneth D Mandl

**Affiliations:** ^4^Department of PediatricsHarvard Medical SchoolBostonMAUSA; ^3^Division of Emergency MedicineChildren’s Hospital BostonBostonMAUSA; ^2^Division of Adolescent MedicineChildren’s Hospital BostonBostonMAUSA; ^1^Children’s Hospital Informatics Program at the Harvard-MIT Division of Health Sciences and TechnologyChildren’s Hospital BostonBostonMAUSA

**Keywords:** Medical records, medical records systems, computerized, personally controlled health records (PCHR), personal health records, electronic health record, human factors, research design, user-centered design, public health informatics

## Abstract

**Background:**

Consumer-centered health information systems that address problems related to fragmented health records and disengaged and disempowered patients are needed, as are information systems that support public health monitoring and research. Personally controlled health records (PCHRs) represent one response to these needs. PCHRs are a special class of personal health records (PHRs) distinguished by the extent to which users control record access and contents. Recently launched PCHR platforms include Google Health, Microsoft’s HealthVault, and the Dossia platform, based on Indivo.

**Objective:**

To understand the acceptability, early impacts, policy, and design requirements of PCHRs in a community-based setting.

**Methods:**

Observational and narrative data relating to acceptability, adoption, and use of a personally controlled health record were collected and analyzed within a formative evaluation of a PCHR demonstration. Subjects were affiliates of a managed care organization run by an urban university in the northeastern United States. Data were collected using focus groups, semi-structured individual interviews, and content review of email communications. Subjects included: n = 20 administrators, clinicians, and institutional stakeholders who participated in pre-deployment group or individual interviews; n = 52 community members who participated in usability testing and/or pre-deployment piloting; and n = 250 subjects who participated in the full demonstration of which n = 81 initiated email communications to troubleshoot problems or provide feedback. All data were formatted as narrative text and coded thematically by two independent analysts using a shared rubric of a priori defined major codes. Sub-themes were identified by analysts using an iterative inductive process. Themes were reviewed within and across research activities (ie, focus group, usability testing, email content review) and triangulated to identify patterns.

**Results:**

Low levels of familiarity with PCHRs were found as were high expectations for capabilities of nascent systems. Perceived value for PCHRs was highest around abilities to co-locate, view, update, and share health information with providers. Expectations were lowest for opportunities to participate in research. Early adopters perceived that PCHR benefits outweighed perceived risks, including those related to inadvertent or intentional information disclosure. Barriers and facilitators at *institutional*, *interpersonal*, and *individual* levels were identified. Endorsement of a dynamic platform model PCHR was evidenced by preferences for embedded searching, linking, and messaging capabilities in PCHRs; by high expectations for within-system tailored communications; and by expectation of linkages between self-report and clinical data.

**Conclusions:**

Low levels of awareness/preparedness and high expectations for PCHRs exist as a potentially problematic pairing. Educational and technical assistance for lay users and providers are critical to meet challenges related to: access to PCHRs, especially among older cohorts; workflow demands and resistance to change among providers; inadequate health and technology literacy; clarification of boundaries and responsibility for ensuring accuracy and integrity of health information across distributed data systems; and understanding confidentiality and privacy risks. Continued demonstration and evaluation of PCHRs is essential to advancing their use.

## Introduction

A personal health record (PHR) is a digital Web-based collection of a patient’s medical history in which copies of medical records, reports about diagnosed medical conditions, medications, vital signs, immunizations, laboratory results, and personal characteristics like age and weight are stored [1]. PHRs have been much discussed over the past few years, and considerable activity concerning them is occurring in health information technology, policy, and market sectors. In recent years, three personally controlled health record (PCHR) platforms have launched: Google Health, Microsoft’s HealthVault, and the Dossia consortium of large employers (including Walmart, Intel, and AT&T) which has a platform based on our open source Indivo PCHR. The PCHRs are a special class of PHRs distinguished by the extent to which users control record access and contents [2]. User control over these functions is governed by subscription and access control mechanisms and annotation capabilities within the record system [3]. It is generally assumed that increasing individuals’ abilities to view and share their medical histories or clinical decision support messages, including from distributed information sources, multiple care sites, and time periods, will result in better self-care preparation and motivation, reductions in treatment and medication errors, and improved health [2-7].

While anticipated benefits of PCHRs may eventually drive their diffusion, the overall approach of a citizen- or patient-centered health record system that interoperates with, but is not tethered to, a provider system represents a fundamental change from current approaches to health information management. Transforming standard practice may be challenging for a myriad of reasons [8]: low levels of health and technology literacy may impede technology uptake and use [9,10], distrust of Web-based health information systems [11], privacy concerns [12-14], and inequalities in access to/accessibility of computerized health information tools may slow adoption [15,16]. Adoption and use may be negatively effected by fears of disrupted and altered service relationships and pushback from providers. Rapid technology development and potential for benefit from PCHRs underscore the importance of understanding acceptability, barriers, and facilitators to their use.

We conducted patient-centered research on beliefs and reactions to the Indivo PCHR, a model PCHR deployed as part of a federally funded technology demonstration. Indivo is an open source PCHR platform that has served as the model for the burgeoning PCHR movement [2,17]. In situ/in vivo experiences and preferences for using PCHRs such as Indivo are important additions to survey and opinion research about PHRs in general. Indivo combines a Web-based health record with integrated capabilities for running a survey tool and rules engine, decision support and health messaging components, user-defined access controls, and sharing and annotation capabilities [2,7]. We deployed Indivo to a community of early PCHR adopters and undertook a range of formative research efforts to learn more about beliefs and behaviors governing PCHR use, stakeholder and lay understanding of the technology, and reactions to the system. Our primary aim was to learn more about the acceptability of PCHRs using Indivo as a test case by describing assumptions about the technology, as well as barriers and facilitators to its adoption. Our secondary aim was to identify design and policy issues germane to best practice technology development for consideration prior to refining the system for diffusion and evaluation.

## Methods

Qualitative data about beliefs, attitudes, and preferences related to the personally controlled health record system were collected over a two-year period (May/June 2006 through April 2008). Questions and observational assessments focused on identifying assumptions, reactions, and preferences germane to the PCHR technology, as well as barriers and facilitators to its use. All study protocols were reviewed and approved by Institutional Review Boards governing human subject research at both the study site and Children’s Hospital Boston.

### Setting

The study was sited in an urban area within the northeastern region of the United States. The setting was a community-based, university health maintenance organization, and the samples, described below, were comprised of persons affiliated with the site and setting.

### Samples

A range of study participants was included in each of three formative research activities: administrative, clinical, and public health stakeholders (n ~ 20) from the study community participated in focus group and one-to-one interviews. Participants were adults, 35 - 60 years of age, with training in medicine and/or health care administration. There were 12 usability testers and 40 pilot participants, all of whom participated in observational assessments and usability and pilot testing antecedent to the system’s demonstration deployment. Testers included undergraduate, graduate, employee, and retiree populations 25 - 65 years of age. Approximately 250 users 18 - 83 years of age (with an average age of 53) participated in the demonstration study, from which 81 email communications to study administrators were logged and their content analyzed. All participants were English speakers, were volunteers, and provided written informed consent and HIPAA authorization for sharing personal health information when piloting with live records.

### Analytic Approach

Narrative data were collected in three formats: (1) transcribed audio-taped focus group interviews conducted with stakeholders and pilot users; (2) written observational notes of usability testing compiled by the study team; and (3) text communication from email exchanges with demonstration participants. Collection and analysis of data for each of these activities involved the following specific activities.

We used a semi-structured protocol to collect focus group data. Questions about health information management, Internet use, and personally controlled health records were asked of early adopters, including administrators and clinicians at the study sites. Follow-up probes were used to elicit information about attitudes, preferences, and reactions to the PCHR model. A trained moderator and an observer facilitated group discussions, and all data were transformed to narrative (transcript) notes for analysis.

We used a formal usability testing protocol to observe interactions with, and reactions to, the PCHR in a semi-standardized fashion. The protocol involved engaging testers in interactions with an advance (beta) version of the final system configured as a live record. Each tester’s record was populated with the test user’s actual medical record data with individual consent and IRB approval. Testers completed specific tasks presented to them in a checklist of test activities (eg, review your laboratory data, add a “device” to your record in the appropriate location). A range of activities was included in the test, including registering, reviewing personal health information, amending health information, identifying categories of information (eg, laboratory results, problems, medications), completing behavioral health surveys, and sharing health information. Testers were observed by a trained team of developers and the study principal investigator who took notes about questions, failures, reactions, level of interest, and engagement. Each observer was assigned 2 - 3 testers to follow in a demonstration setting. Testers were asked to “talk aloud” their thoughts and actions as they completed the various team-specified functions. The objective was to learn more about how they interacted with the system to solve problems and to assess whether attempted actions matched expectations. At the close of testing sessions, semi-structured discussions were held to elicit feedback from testers. Notes made by observers were compiled as memos in narrative form which were used to fine tune the user interface and inform our understanding of reactions to the technology and individual engagement with personal health information.

We tracked all subject-initiated email communication with the study team through the full pilot period of six months. Emails were individually reviewed, redacted of identifying information, organized, and analyzed for major and minor themes as described above. Two independent, trained analysts reviewed communications, independently coded them, and then reviewed their summaries to arrive at a consensus. Narrative data were summarized for this report.

For analysis of all narrative/text data, two analysts (ERW and LK) worked independently with a shared rubric of major thematic codes to describe the data. Major constructs were operationally defined for thematic analysis as follows: *Awareness of PHRs/PCHRs* was defined as familiarity with the concept and/or practice of Web-based, patient-controlled health record systems. PCHRs were distinguished from PHRs and electronic medical records by the degree to which patients versus providers have control over the system and its information content. *Privacy of personal health information* was defined as the ability of individuals to control access to their PCHR and the security/integrity of health information [18,19]. *Autonomy* was operationally defined as individual control over selection and subscription to data sources; the ability to self-report or update health information; authorization of access and sharing permissions; and control over messaging. Additionally, several pilot users were debriefed about the consent process, considered a key component to the public face of the PCHR [20]. Analysts read all narrative data independently to assign codes to text fragments and develop subsidiary coding schemes. Coding schemes and transcripts were worked iteratively and inductively to refine them and achieve consensus. Data were triangulated across the three assessment activities to build a comprehensive picture of issues related to awareness, privacy, autonomy, and barriers to/facilitators for acceptability and use.

Findings reflect triangulation of data collected at different junctures over the formative evaluation and pilot period but do not reflect a pre/post assessment of acceptability. We report on experiences and perceptions common across multiple respondents. Illustrative quotations are provided in table form (with select exceptions where quotations are included in the descriptive text) to describe prevailing norms and experiences. Barriers and facilitators were identified by the study team, based on close observation of the pilot system implementation in the context of other formative data, and reflect the consensus of the study team, drawing on a synthesis of stakeholder and user reports and experiences generated from the data. Barriers and facilitators are categorized as primarily societal, interpersonal, or individual level factors. Practice recommendations for policy and design are suggested in the discussion section and summarized in a text box based on the pattern of observed barriers and facilitators and formative findings.

## Results

Findings which concern levels of awareness of PCHRs, privacy, and autonomy, as well as variations by stakeholder group/role or age group are summarized below and discussed (Table 1).

### Awareness of PCHRs

Participants demonstrated low levels of awareness about personal health record technologies including PCHRs. Prior to the pilot deployment, none were using automated systems to store or manage their own health information, and none had heard about or followed public or professional discussions about PCHRs. No age differences were evident in awareness about PCHR technology in general. Variation in understanding about subscription models to sources of personal health information (PHI) may have been present. Younger individuals and students (ie, those in the 18 - 35 approximate age range) appeared more familiar with the concept of subscribing to a data system; however, few subjects appeared to have considered this model for obtaining personal health information.

Overall, participants appeared to overestimate the extent to which personal health information is available and flowing electronically within provider systems. Many assumed that such information flow already occurs or that it was inevitable in the near future. Perceiving oneself as personally excluded from electronic health information systems was common. Desire for inclusion and control over PHI comprised a significant motivator for system adoption/use. No differences in age were apparent in these beliefs and perceptions.

### Privacy

In general, we observed a moderate level of concern about privacy characterized by a pragmatic technology-supporting norm in which risks to privacy were considered unavoidable. Several specific mechanisms by which privacy might be threatened were identified, including: intentional identify theft, disclosure and misuse of information by insurance companies, accidental mix-up of records and their contents, mismatch of medical records data with personal health records, and misuse and inappropriate viewing, including “snooping” and attempts by health professionals to track or follow-up on outcomes of former patients and co-workers. Participants recognized the potential that privacy could be breached and that such breaches could result in serious harm. The most salient adverse outcomes related to breach of privacy were threats posed to insurability and/or denial of employment or care. Across all groups of subjects, the possibility of an audit check appeared to be among the most reassuring and accessible options for safeguarding privacy and building confidence.

Perceived risks to privacy were offset by an understanding that one’s privacy is risked in paper information exchange as well. Concerns about inadvertent or intentional breach of privacy were discounted by the high value placed on ready access to health information, especially in emergency conditions.

Students and younger users (typically those under 50) may be more sophisticated than older users about technological strategies for ensuring privacy. They appeared familiar with a range of technologies to improve privacy and security including use of encryption, digital signatures, and certificates. Despite their greater technological sophistication, younger users may possess a limited understanding of harmful consequences of sharing information and maintaining a lax privacy practice. In focus groups, young adult subjects (18 - 35 years of age) expressed widely varying opinions about whether it was safe to share health information with persons who were not providers; moreover, younger individuals appeared to be poorly informed of, and to have a naïve appreciation for, potential risks to insurability or employability related to disclosure of health information/records and problems.

Older and retired participants perceived risks related to a breach of privacy and reported they have “less to lose” than younger and employed persons. Some participants expressed concern about stigma or discrimination resulting from the release of PHI. Risks from inadvertent release of infectious, mental health, and chronic disease diagnoses were all recognized, with no clear emphasis on one category of illness as being particularly problematic. Participants, especially older ones, were worried that information disclosure through a PCHR could impose an emotional burden on family members.

Across age groups, many individuals assumed that sick individuals would be acutely concerned about privacy risks and less willing to participate in electronic information exchange/data sharing than healthy individuals. The assumption that sick persons are more concerned than healthy persons with privacy was not borne out in preliminary findings. For some users, chronic illness appeared to offset perceived risks associated with information sharing and motivated demand for accessible and transmissible information, as indicated by one participant:

I would be very interested in having access to all my records. I think this type of program will make my life in particular a whole lot easier.

Within the pilot, concerns for privacy were rapidly overridden by the need for help understanding technical or clinical issues. Participants readily disclosed personal information about diagnoses, conditions, and complications with project staff through email communication in the context of verifying and understanding information in their record:

Hello, I'd like some explanation of the health record that has been posted on my indivo page. There are things there I've never heard of, and important things that aren't there. I don't know what anything means .... Something says I was screened for malignant cancer of the cervix. When? I don't remember anything like that. And viral arthritis? When? What? Please explain, please refer to me to someone who's in charge.

Some participants shared nonclinical, identifying information, including passwords, in email exchanges with project staff. Actual privacy practices were different from espoused privacy concerns for some participants, and substantial vulnerability to privacy risks was observed.

### Autonomy

Users expressed high value and interest in the concept of autonomy and welcomed greater access and control of their health information. While highly valued, autonomy was perceived as a double-edged sword. Sticking points included concerns about the locus of responsibility for maintaining the accuracy and integrity of PHI. Users wanted assurances that outdated or erroneous information that they identified and amended in their personal health record would be updated in subscription data sources. They were concerned about ramifications of intentionally or inadvertently changing PCHR contents and nervous about entering their own information using the system’s tools for annotation. Discomfort among some users with the idea of personal or patient annotation was echoed by providers and health service administrators who framed this concern in terms of quality of care, completeness of health information, and risks for liability.

While individual control over PHI was valued highly by younger/student participants (18 - 35 years of age), substantial variability was evident in opinions about the safety of granting write access control over their records to any other person, including for some, a primary care provider. Some viewed the patient/record owner as the final arbiter of a record’s contents, while others considered the primary care provider as the final arbiter. The value older participants assigned to personal control and autonomy, including as a source of accessible information in emergency conditions, was mitigated by the concern that their records could be inaccessible should they become impaired due to illness or age if they did not arrange for access by significant others or proxies.

Generally speaking, users placed a premium on the ability to control access to their health information and, generally speaking, favorably viewed options to control access to their PHI and share their records with members of their family or close social group (significant others, etc). Nevertheless, few users formally shared their records in practice despite the ability to do so; those that did used workarounds or informal—and riskier—approaches to sharing, rather than the formal sharing mechanism engineered within the system. The pilot system was implemented with a model of strict individual autonomy. This model was intentionally subverted by several users who shared passwords and account information with family members to advance caring relationships. Evidence of this turned up throughout the pilot as sharing of email accounts and record information, typically among older spouses as multiple email communications illustrate (Table 1).

Strict user control of incoming and outgoing messages delivered through the PCHR was viewed by many participants as an essential ingredient of an autonomous system and a prerequisite to sustained use. The ability to filter incoming messages by content and frequency was highly valued. Such abilities may be inconsistent with expectations of automatic tailoring of messages to contents of records and prior health communications. Vertical integration of systems such that messages, alerts, and communications are wholly integrated with user preferences represent design/implementation areas for which tools and best practices may be needed.

**Table 1:** Awareness, privacy, and autonomy factors bearing on acceptability of personally controlled health records

**Construct**	**Finding**	**Age/role pattern**	**Illustrative quotations**
**Awareness**			
Awareness of electronic medical (health) records	Assumption that health information is digital, ubiquitous, accessible	All groups	*[Personal health] information is more and more on computers ... whether I choose to interact with that doesn’t change the fact it’s online and everyone else is interacting with it ... the only choice I’m making is whether I interact with it.*
Access to electronic health records, PHI	Perceived exclusion of individual access to electronic PHI	All groups	*The truth is that in terms of our access, we’re the only one’s that don’t have it. In terms of my life all my information is electronic. We’re the only ones who don’t have it: How crazy is that?*
Familiarity with patient-controlled health record systems	No prior use or familiarity, intrigued and assume PCHR will advance quickly	All groups	*I think ten years from now we won’t even be discussing this, five years from now ... it’ll be a done deal. Five years from now ...*
**Privacy**			
Ability of individuals to control access to their PCHR and the security/integrity of health information	Moderate concern, pragmatic, technology supporting norm	Young adults naïve to risks from sharing	*The systems will continue to do what they can to maintain [privacy] and the reality of our world is that in some cases as we see in the papers all the time privacy will be breached. And that’s part of the reality of the world we live in and continue to live in and the choice we make [to interact with technology and use systems] has nothing to do with that.*
Perceived privacy risks and threats	Concern for abuse of information by insurer, employer	Greater among employed	*Is there anyone who is going to be able to access that information who is going to be damaging to me who is going to use that information in a bad way—an insurance company who can have access to the information anyway and always have?*
			*The thing I want to be hesitant with, it’s kind of a moral issue with a future employer maybe, don’t want to see that I’ve been tested for diabetes and the amount of family history of diabetes ... because they’re afraid that I night die when I’m 30 or 40 and they might want to hire me forever ...*
	Assumption that persons with health problems more vulnerable, more concerned	All groups	*On the medical side, having information online, having it shared, I perceive that as a personal benefit ... If I go to one physician/system then to another, that doctor can pull up my information ... I view that as a personal benefit and I want that for my own health. If I had a sensitive health issue or diagnosis, I might view it differently ...*
			*If I had a sensitive med problem might have more worry about [privacy breach, sharing information] ...*
Perceived qualifiers of privacy risk	Understanding that other information media (including paper records) have risks	Greater among administrators and providers	*I think you are more vulnerable with a paper record. I have seen more errors with paper records, papers misfiled and you see that in a paper record. It’s human error. It’s usually that the MRN is one digit off ... Is that not a breach of confidentiality? Or, you take pieces of information and put them in an envelope and send it to medical records. That’s not very secure if you ask me!*
	Perceived personal benefit of access to health information	All groups	*[It’s] to a consumer’s advantage to have that information shared by all your providers and to be able to access it yourself to some extent.*
			*I think on the medical side, having the info online, having it networked with the hospitals I go to, I perceive it as a personal benefit, I’m going to benefit.*
Safeguards against risk	Premium value on audit check	All groups	*But the thing is there’s an audit. On a paper [record], there is no audit.*
			*I can understand how it puts the patient in control of who sees his/her records, but I want to understand that there is a clear and easy-to-use means of monitoring who has access at any given time and the patient has the ability to change that permission at will.*
**Autonomy**			
Control over subscription, self-reporting, sharing, messaging	Favorable view of autonomy	All groups	*I like the “out-of-the-box” concept of putting the patient in charge via their own control of the records.*
			*You know I kind of think about this as ... when I have a mammogram or couple months ago I had an MRI, I don’t want a report from the doctor that says “it’s normal” I want “the report”. So what I have to do is I have to call, then I have to fax them an okay, then they won’t fax it to me ... they have to mail it to me. To me, it’s because I want it, it’s not their legal obligation to send me a copy of that report. It’s their ethical and practice obligation to let me know the results. So I kind of think about this online, record online, it’s my record, it’s nobody else’s record, if I want this [report] in it, it’s my choice. I might say that I don’t want my neurologist or whatever to put anything in to it.*
	Concern about quality, accuracy and locus of responsibility for maintaining record, workflow impacts	Shared by patient, administrator, provider	*[W]hat becomes our responsibility here in terms of patient care? Let’s say something goes really bad with a patient and it turns out that there’s a piece of information in the PHR that if our clinician had had access to it or had been looking closely at both records, the outcome could have been different ...* [administrator]
			*If the patient has their own record, there is a lot of information they don’t understand, there could be a lot of phone calls to their provider to explain the information that they don’t understand. And there will be a lot of phone calls to their physician to explain. And we can’t fit in a visit [to the clinical calendar] to explain ...* [provider]
			*So as a provider, if I look at it, I have to look at it for what it is: the information that’s in there is what the patient wants in there, and there may be other variables, that it’s not all there.* [provider]
			*I have checked my record and the latest two years of immunizations are still missing. There is a window where I can add them but that seems not to be in the spirit of the system. It would be better if such info were added by someone authorized who has the correct data.* [patient]
			*Apparently I cannot edit the medications in my record and there are errors. I've added annotations, but either I should be able to edit the record, or there should be some way for me to get corrections made.* [patient]
	Uncertainty about appropriate and safe read/edit access policies	Evident among young adults/students	*[What kind of access would you give primary care provider?] Read, append, not necessarily write, [primary care providers] don’t get to delete things…*
			*It would be nice if the physician could delete [information] ... if you update something*.
			*You should have final say over what’s in your record ...*
			*No one should be able to delete something in a record ...*
	Concern about aging, illness and competency to manage records	Evident among older users and retirees	*... Finally, you will have to prepare for the final insult where someone capable of using the system becomes incapacitated and the system still needs the records even though the password and permission is locked in a non-responsive being (accident trauma, Alzheimer disease, etc).*
	Subversion of strict autonomy controls by users in caring social relationships	Evident among older users	*Hi - I answered the Indivo Nov. 15 survey just now. However, it came to our Verizon email address instead of the email address that I use. My name is ____, user name _____ and the email I use is __. The survey came to the email address that (spouse) uses, and he was unable to log in using his user name of _____. We decided it was for me - do you have the correct email addresses for each of us?*
			*My wife's account and mine are overlapping and while she can access her site with you, mine has her name and address listed for me. Is there a way of separating them?*
	Preference for strict personal control of messaging	All groups	*I would like to control this system so that I receive ONLY specific items [messages] related to my PERSONAL health specifics.*

### Barriers and Facilitators

Barriers and facilitators to adoption and use of the system were identified at institutional, interpersonal, and individual levels from across all formative data collected (Table 2). Several of these barriers and facilitators were notable for their broad policy and practice implications and are highlighted here.

Uncertainty about locus and extent of responsibility for ensuring PCHR accuracy given the distributed nature of subscription data sources comprised a barrier to adoption across social levels. Concern was expressed by administrators, clinicians, and patients/individuals about potential liability and quality of care risks associated with patient access to PHI through a clinically disintermediated system that permits user annotation and sharing. The potential for confusion and misalignment of information systems resulting from diverging health information in cases where users annotate or amend patient-reported information in the PCHR was noted. The absence of a mechanism for automatically feeding back annotations to subscription data systems concerned stakeholders from all groups. Concern over this issue may comprise an impediment to adoption at institutional and individual levels.

In a similar fashion, uncertainty about responsibility for clarifying the meaning and contents of records and concern about time requirements to address patient questions affected stakeholder buy-in and challenged norms for interpersonal relationships between patients and providers. While observed levels of problems were lower than anticipated, they were exacerbated by gaps in health and technology literacy. Older/retired persons in particular encountered technical barriers around system access, underscoring the importance of clarifying responsibility and resources for help. Lay and technical vocabularies do not match, causing consternation among users who see unfamiliar and/or frightening content in records. Providers are not always well positioned or resourced to respond to users’ questions.

Our PCHR system was available as part of an IRB reviewed demonstration. A dearth of guidelines and precedents for operating human subject research within a PCHR environment posed barriers to implementation and required education of IRBs and review panels. Research norms stipulating tight investigator control of information are contradicted by PCHR models of strict individual autonomy and control of information. The tension between these models needs to be understood and negotiated with IRBs to authorize research-based implementations. We developed and used an automated multistage consent process that included authorization for release of health information as stipulated under the Health Insurance Portability and Accountability Act [19] to alert and educate users to the significance of PCHR-enabled health information exchange. The multistage consent protocol was partitioned into: a first screen that provided summative information about the study and consent; a second screen that included the full consent and HIPAA authorization; and a third screen with a point-by-point affirmation of consent elements and electronic signature. Despite perceiving that the multistage consent process was arduous, users endorsed its length and the sequential conditioning of information as, “telling me something serious was happening” and “letting me know that you [system/investigators] take this approach seriously”. IRB review and a multistage consent process appeared to facilitate lay participation and trust in our research demonstration.

Workflow planning and organizational will are required to ensure appropriate effort is given to authenticating users. From the perspective of institutional gatekeepers and stakeholders, building an interoperable bridge with a vendor-based heath information system to subscribe to EMR data required a modest commitment of resources and had a minimal impact on deployment/use. A modest burden was experienced around authenticating participants/users and development to ensure appropriate export of data from source EMRs to PCHRs.

On the other hand, close alignment of the system with trends for consumer-centered health care and information systems facilitated acceptability at the institutional level and primed acceptance for the approach among some users [21]. The perceived value of the system for advancing knowledge and supporting care and the noncommercial nature of the project facilitated buy-in and participation at institutional and individual levels. The value of a patient reporting to a record prior to a medical visit in order to support care and optimize time was highly valued: institutions, patients, and providers all understand time and attention limits around care visits. The potential value of using a PCHR to support efficient use of a limited resource facilitated acceptance. Institutional stakeholders and users readily identified assets of the PCHR approach relative to portals, especially with respect to the suitability of PCHRs for maintaining life-long health information, traveling with individuals as they leave care settings, and supporting “family” records and socially embedded caring relationships.

Finally, the value of using the PCHR as a platform for increasing health literacy and health engagement was evident in feedback from participants provided during usability testing and communications with the study team, and this may facilitate future development. Users were keenly interested in having a personally controlled health record and in the possibility of the technology advancing toward a platform model that supported multiple functions, including user interface functions that would allow mouse-over explanations of medications, drill-down capabilities to investigate treatments, definitions of medical conditions, problems and treatment strategies, summaries of research evidence, and even—among some testers—linkages to research data. Similar interest was expressed in applications supporting personalized feedback and contextualization of health information, including support for individually reported survey/annotations collected within the PCHR. Interest in these functions was evident across user groups but was consistently expressed by younger (primarily student) users and working adults.

**Table 2:** Barriers and facilitators to adoption and use of a personally controlled health record system

Barriers	Facilitators
**Societal level factors**	
Poorly defined locus of responsibility for ensuring information accuracy, integrity	Perceived alignment of PCHRs with norms, trends for consumer-centered health care and information systems
Administrative concerns about liability risks if patient record more complete than provider record	Institutional prioritization of HIT to advance health care and communications
Concern about workflow impacts on IT and clinical staff	Stakeholder support for community participatory research
Complications of interoperating with an evolving vendor-based EMR development landscape	“Branding” of test system and study materials as originating from IRB governed study conducted by a trusted nonprofit
Absence of clear policy/practice supports guiding PCHR use including for research and associated human subject guidelines	Stringent data security: storage behind firewalled, individual record encryption, certificate authentication system
Lack of a private, unique identifier for patients	
**Interpersonal level factors**	
Provider resistance to allowing patients record access	Outreach to participants from trusted clinical staff at the site
Insufficient time for providers to participate in collaborative record review and address questions from patients about record contents	Perceived utility of a system that allows reporting about health behaviors to a record prior to a provider visit to optimize visit time
Concern that PCHR will challenge provider/patient roles, relationships and that providers will be uncomfortable sharing power	Utility of PCHR "family" record model for supporting health throughout families and across generations
	Perceived utility of PCHRs for sharing information among providers in multiple locations to facilitate comprehensive care.
**Individual level factors**	
Low levels of technological literacy, self-efficacy especially among older cohorts	Technological know how, experience with other individually controlled record systems (ie, banking)
Uncertainty about who is responsible for ensuring information accuracy and integrity: hesitation, low self-efficacy in navigating health information	Experience with a chronic health problem or need for greater/easier access to a family member's health information
Distrust of Web-based health systems and IT	Attitudes favorable to individual control and autonomy
	Discounted worry about consequences of a privacy breach by users who see value in access to information

## Discussion

### Principal Results

Formative evaluation about acceptability of a PCHR in a community setting confirmed that issues related to privacy, autonomy, and accessibility of technology and health information all play a role in uptake and use of nascent systems. Low levels of awareness about personal health record technologies, including PCHRs, and lack of familiarity with the concept of subscribing to a health information data source may produce barriers to creating robust and complete records for some users. Keen privacy concerns coexist with pragmatic norms when addressing the risk of privacy. These factors were identified within the context of low levels of awareness about PCHR technologies and substantial thirst for access to, and control of, PHI. Privacy and safety conditions prefigured trust and were lynchpins for technology adoption and use, consistent with expectations [12]. Espoused concerns for privacy were belied by somewhat lax privacy practices, indicating a need for careful design-based protections in which users are continually educated and reminded about safe practice. This may be especially so among younger individuals whose privacy concerns may be naïve. The self-selected nature of the pilot sample precludes assessment of the degree to which privacy concerns impeded technology uptake. However, we saw little indication that privacy concerns alone constitute a barrier sufficiently large to impede broad adoption and use.

Strict protection of autonomy was highly valued among PCHR users. Nevertheless, autonomy practices were intentionally subverted within some family and social relationships consistent with others’ reports about management of health information in the home [23]. Perceived imperatives to solve technical problems and/or understand the meaning of health information rapidly eroded privacy and autonomy practices among users. Users readily disclosed personal information and gave others access to their records to solve technical problems or discuss record contents. Expressions of uncertainty about the locus of responsibility for verifying the accuracy of PCHR contents and for ensuring alignment of distributed health information systems where users could annotate the PCHR were voiced by all stakeholder groups and reflect tensions relating to the autonomous PCHR model.

Sharing capabilities were highly valued but underutilized in this early deployment. Findings confirm predictions of the needs for technical assistance and for education of users engaged with this new approach to information [24]. Assessment of, and planning for, the effects of broad technology diffusion on the workflows of a range of stakeholder groups is needed: impacts of this new approach on clinicians, information technology professionals, and staff providing ethical oversight and management of HIT and research need monitoring. Clear operational guidelines, governance systems, and administrative supports are needed, along with relevant consent and technical assistance materials. Caution is warranted when basing PCHR policy and design decisions on opinions about privacy and autonomy without practice-based evaluation, given the possibility for divergence between policy and practice and distortion of others’ preferences and sensitivities.

### Implications for Policy and Practice

The following summative conclusions for design and policy work to advance PCHRs are offered based on observations from formative research relating to a first community-based deployment of an integrated PCHR:

- Discussion about technical and policy approaches is needed to identify strategies for aligning PCHR and subscription data systems, as feasible, to address the possibility of misalignment of information systems where individuals can amend/annotate patient-reported information in the PCHR. Discussion about design options that allow feedback, flagging or reciprocal notification of amended patient-reported information in the source record may be useful given concerns about misalignment and attendant risks for misinformation across individual user, administrator, and clinician groups. The acuity of this issue may increase with intensifying federal emphasis on rapid advance of PCHRs. In the scenario where the PCHR becomes the “record of record”, problems of alignment may be resolved.

- Clear lines of responsibility and dedicated resources are needed to support users and advance their understanding of the contents of their records and the meaning of health information to maximize gains from PCHRs. Gaps in health literacy may eventually diminish as the technology proliferates in the emerging marketplace of health information supports. However, discussion and testing of design-based mechanisms for addressing gaps, for example through mouse-over and drill-down capabilities, is warranted, as are exploration and possibly resource allocation for supports extrinsic to the technology that may serve less literate populations. These may include health coaches, interpreters and guidance staff, and/or technology/information system navigators. Consideration of the possible cost and benefit of supporting new ancillary staff positions may be needed in light of the possibility of interrupted or increased clinician workflow when/if users seek more information about their health and records.

- Clear guidelines and materials to educate users about this new technology, privacy risks and safety mechanisms, including those pertaining to sharing approaches are needed. Social marketing materials may be required to advance technology use, clarify dangers, and address barriers of trust and understanding and reduce potential for abuse. Consideration of demographic differences in need may be required: younger users may be especially naïve to adverse consequences of sharing health information, given their norms for intensive information system use and sharing through electronic social media; older users may face greater barriers related to technology literacy in general and special needs around understanding issues related to competency, proxies, and sharing across generations.
- Creation of a family-focused health record system, seen by many as a logical extension of the PCHR approach [22], is not yet supported by a clear technology and practice model. Subversion by users of strict individual autonomy models for PCHRs suggest the merits and relevance of exploring whether and how personally controlled health records can be designed and rooted in policies that reflect options for social and familial records to support caring relationships and collective knowledge.
- Advances in protocols and models for governing human subject participation in research-based PCHRs and research which enables, or operates through, PCHRs are needed. Education, training, and technical assistance materials are necessary for investigators, IRB panel members and offices, and individuals/subjects. Parallel mechanisms and supports are needed for monitoring fairness, safety, and transparency in commercial and service PCHR applications. For the latter, IRB oversight and consent may not be required although mechanisms for clarifying terms of use, information control, governance, quality assurance, and health information exchange are needed.

### Limitations

To the best of our knowledge, this is the first report about the acceptability to users in a community-based setting of a personally controlled health record—in this case, a platform system that puts users in control of PHI from an electronic medical record to which they subscribed. This study was sited in a community of early adopters with relatively high levels of health and technology literacy. This work is limited by its single site/early adopter design and inherent selection effects stemming from that design. Continued study is needed which employs approaches representative of the population and standardized metrics to explore the potential for variations in PCHR acceptability, and barriers and facilitators which reflect age, role, and other characteristics suggested by findings of this formative work. The work is also limited by the early stage of technology development of the test system and the qualitative methods employed. These factors limit inferences about the broader acceptability of the PCHR technology and impact of various barriers/facilitators. Such limitations are, however, typical in formative research of a new technology or concept. Rigorous evaluation of PCHR deployments in expanded samples/settings are recommended to advance understanding of PCHR acceptability and impacts and to develop best practice approaches for addressing the institutional, interpersonal, and individual challenges.

### Conclusions

PCHRs are widely viewed as a disruptive innovation that may be transformative in health care. Before we can expect uptake by consumers en masse, potential barriers to adoption and use must be addressed. Early experiences with Indivo, the original reference PCHR, have identified societal, interpersonal, and individual level barriers and facilitators to address through near term system redesign and revised social marketing of the technology. Responding to these observations and continued evaluation may substantially advance the use and relevance of the PCHR platform model otherwise endorsed by users.

## Abbreviations

EMR: electronic medical record
HIPAA: Health Insurance Portability and Accountability Act
HIT: health information technology
IRB: institutional review board
PCHR: personally controlled health record
PHI: personal health information
PHR: personal health record
